# Interleukin-41: a novel serum marker for the diagnosis of alpha-fetoprotein-negative hepatocellular carcinoma

**DOI:** 10.3389/fonc.2024.1408584

**Published:** 2024-05-21

**Authors:** Yazhao Li, Haoyu Wang, Danfeng Ren, Jingyu Li, Zihan Mu, Chaoyi Li, Yongchao He, Jiayi Zhang, Rui Fan, Jiayuan Yin, Jiaojiao Su, Yinli He, Bowen Yao

**Affiliations:** ^1^ Center for Translational Medicine, The First Affiliated Hospital of Xi’an Jiaotong University, Xi’an, China; ^2^ Zonglian College, Xi’an Jiaotong University Health Science Center, Xi’an, China; ^3^ Department of Communicable Disease, The First Affiliated Hospital of Xi’an Jiaotong University, Xi’an, China; ^4^ Biobank, The First Affiliated Hospital of Xi’an Jiaotong University, Xi’an, China; ^5^ Department of Hepatobiliary Surgery, The First Affiliated Hospital of Xi’an Jiaotong University, Xi’an, China

**Keywords:** IL41, METRNL, hepatocellular carcinoma, AFP, serum biomarker

## Abstract

**Background:**

For the lack of effective serum markers for hepatocellular carcinoma(HCC) diagnosis, it is difficult to detect liver cancer and identify its recurrence early.

**Methods:**

Databases were used to analyze the genes potentially associated with alpha-fetoprotein(AFP). ELISA assay was used to detect the serum IL-41 in HCC, liver metastases, hepatitis, and healthy people. Immunohistochemical staining was used to analyze the relative quantification of IL-41 in HCC and paracancer tissues. Various survival curves were plotted according to clinical pathological data and helped us draw the ROC curve of IL-41 diagnosis of HCC.

**Results:**

The serum expression of IL-41 was highest in AFP negative HCC patients and significantly higher than that in AFP positive HCC and metastatic cancer patients. There was a significant negative correlation between elevated serum IL-41 and AFP(<1500ng/ml). The clinicopathological features suggested that the serum IL-41 level was significantly correlated with capsule invasion, low differentiation and AFP. High serum expression of IL-41 suggests poorer survival and earlier recurrence after resection, and IL-41 upregulated in patients with early recurrence and death. The expression of IL-41 was higher in HCC tissues of patients with multiple tumors or microvascular invasion. The ROC curve showed that serum IL-41 had a sensitivity of 90.17 for HCC and a sensitivity of 96.63 for AFP-negative HCC, while the specificity was higher than 61%.

**Conclusion:**

IL-41 in serum and tissue suggests poor prognosis and postoperative recurrence in HCC patients and could be a new serum diagnostic marker for AFP negative patients.

## Introduction

Hepatocellular carcinoma (HCC) incidence in China is high (1), with about 50% of global cases originating from China (2). At present, HCC is the fourth common malignancy and second cause of death in China, posing a serious threat to people’s life and health (3). The etiology of HCC is complex and diverse and has been shown to be associated with cirrhosis, viral hepatitis, alcohol consumption, and fatty liver (4). In particular, chronic hepatitis B virus-related cirrhosis was reported as the primary risk factor for HCC development in China (5). HCC progression is a multifactorial, multistep process driven by epithelial-mesenchymal transition (6), the tumor microenvironment (7), cancer stem cells (8), and aging (9). Due to the rapid progression and poor prognosis of HCC, postoperative recurrence remains a major challenge in the clinical management of the disease. Surgery is currently the main treatment approach for HCC (10), but the 5-year postoperative recurrence rate is close to 70% (11). In addition, micrometastases are often difficult to detect by imaging after surgery, resulting in delayed treatment for many patients (12). Therefore, there is an urgent need to identify novel diagnostic markers and therapeutic targets for HCC.

Alpha fetoprotein (AFP) is the most widely used serological marker for HCC worldwide. Its application stems from the discovery that some HCC secretes high levels of AFP (13). In addition, an AFP concentration of 400 ng/mL was recommended as the threshold for auxiliary diagnosis in the 2001 and 2017 Chinese HCC diagnostic staging criteria (14). However, some HCC patients with AFP levels below the diagnostic threshold (e.g., AFP-negative patients) can only rely on imaging for the detection of postoperative recurrence, which often leads to delayed diagnosis and treatment (15). Therefore, novel sensitive markers are needed for identifying AFP-negative HCC patients. Although the combined use of AFP and several serological markers such as DCP, AFP-L3, and PIVKA-II (16) have been shown to improve HCC detection rate, the specificity and sensitivity of these markers are inadequate to provide early HCC diagnosis. Furthermore, efforts in finding serum diagnostic markers for HCC have lessened in recent years, and the serum markers tested in combination with AFP thus far have not resulted in much improvement in sensitivity and specificity. For example, DCP has a 51.7% sensitivity and 86.7% specificity for HCC, while DCP combined with AFP has a 78.3% sensitivity for HCC (17). Large cohort clinical studies conducted at different centers suggest that diagnostic models are superior to serum markers for HCC diagnosis, but their widespread application in the real world is constrained by the incorporation of complex indicators and high detection cost. Another example is the classical liver cancer histological marker HSP90, which has been confirmed to be associated with cancer occurrence. However, since HSP90 is highly expressed in various cancers, such as salivary gland and breast cancers, it is not a specific diagnostic marker for HCC (18).

Interleukin (IL)-41, also known as meteroin-like protein (METRNL), is a newly discovered immunomodulatory cytokine or adipokine expressed in a wide range of cells and tissues (19, 20), most prominently in human subcutaneous white adipose tissue (21). IL-41 has been reported to antagonize insulin resistance (22), and its expression is upregulated by factors such as inflammation, exercise, and cold exposure (23). In addition, IL-41 plays a key anti-inflammatory role in several inflammatory diseases such as psoriatic arthritis and inflammatory bowel disease (24–26). IL-41 can regulate cytokine levels through macrophages and is involved in the modulation of inflammatory responses in a mouse model of sepsis (27, 28). However, the role and mechanism of IL-41 in cancer are currently unclear. This study suggests for the first time that IL-41 is a novel marker for HCC detection and may play an important role in predicting the prognosis and treatment response of HCC.

## Methods

### Patients and clinicopathological features

Among the 176 HCC patients included in this study, 88 were AFP-positive and 85 were AFP-negative. Additionally, 18 CRC patients with liver metastases, 15 patients with acute AFP-positive hepatitis, and 19 healthy controls were also included. All patients received treatment at the First Affiliated Hospital of Xi’an Jiaotong University between 2018 and 2021. The study was approved by the Ethics Committee of Xi’an Jiaotong University, and tissue and serum samples were collected in accordance with medical research ethics. Patient information, including gender, age, tumor size, tumor number, tumor stage, tumor differentiation, capsule integrity, microvascular invasion, and tumor recurrence, were obtained from the electronic medical records of the First Affiliated Hospital of Xi’an Jiaotong University. All study participants included in the data analysis were followed for at least three years up until February 14, 2024. Prognostic survival was assessed using overall survival (OS), recurrence-free survival (RFS), progression-free survival (PFS), and time to tumor recurrence (TTR). OS is defined as the time from HCC diagnosis to death of any cause. RFS is the time from tumor diagnosis to either tumor recurrence or tumor-related death, whichever occurs earlier. PFS refers to the time from randomization to either tumor progression or death of any cause, whichever occurs earlier. TTR is defined as the time from tumor diagnosis to tumor recurrence. A total of 162 patients were included in the analysis, excluding those who had incomplete pathological data or were lost to follow-up, and were divided into the high IL-41 expression (≥ 65.853 pg/mL, median serum expression of IL41) group and low IL-41 expression (< 65.853 pg/mL) group to assess differences in OS, PFS, RFS, and TTR. AFP is considered negative when diagnosing liver cancer with an AFP of less than 20ng/ml. The correlation between serum IL-41 expression and various liver function markers, including serum alanine aminotransferase (AST), alanine aminase (ALT), total bilirubin (TBIL), direct bilirubin (DBIL), total protein (TP), albumin (ALB), platelet (PLT), hepatitis B virus surface antigen (HBsAg), as well as prothrombin time (PT) and activated partial prothrombin time (APTT), was analyzed.

### ELISA

Serum concentration of human IL-41 was measured using the Human Interleukin-41 (IL-41) Quantitative Assay Kit (RX100486). Briefly, high-affinity microplate was coated with anti-human IL-41 monoclonal antibody, incubated with diluted serum samples (1:5) and standards, washed, incubated with biotinylated anti-hIL-41 detection antibody, washed, incubated with horseradish peroxidase-labeled streptavidin (streptavidin-HRP), washed, and incubated with the chromogenic substrate TMB. The reaction was terminated by the addition of a stop solution, and absorbance at 450 nm was measured using a microplate reader. The standard curve was generated by plotting the concentrations of the standards on the x-axis (6 standards plus one blank, totaling 7 concentration points) and the corresponding OD values on the y-axis, followed by a four-parameter Logistic curve fitting. IL-41 concentrations in the serum samples were determined by the sample OD values using the standard curve.

### Immunohistochemistry

Tissue IL-41 expression levels in HCC patients and healthy controls were quantified by IHC. Briefly, formalin-fixed, paraffin-embedded tissues were cut into 2 μm thick sections, cleared by xylene, dehydrated in ethanol gradient, dewaxed in water, washed with distilled water, incubated with 3% H_2_O_2_ at room temperature for 10 min to quench endogenous peroxidase, and washed with PBS 3 times at 5 min each wash. Antigen retrieval was performed using preheated sodium citrate-EDTA solution, and the tissue sections were then blocked with 5% BSA solution at 37°C for 30 min. After tapping off the solution, the tissue sections were incubated with drops of diluted primary antibody (Immunoway, METRL rabbit pAb, YT7556) at 4°C overnight, washed 3 times in PBS (5 min per wash), incubated with biotinylated goat anti-rabbit IgG (BASTER, Wuhan, China) at 37°C for 30 min, washed 3 times in PBS (5 min per wash), incubated with streptavidin-biotin complex at 37°C for 30min, washed 3 times in PBS (5 min per wash), and stained with DAB. After rinsing thoroughly under tap water, the tissue sections were counterstained with hematoxylin and mounted with neutral gum and water-soluble sealant.

The degree of positive IHC staining is influenced by antigen content, distribution, labeling method, and sensitivity. The average gray value (staining intensity) and percentage of positive area (staining area) were quantified by ImageJ and scored as highly positive (3 points), moderately positive (2 points), weakly positive (1 point), and negative (0 point). The total score of IHC is the sum of the percentage of positive area multiplied by the corresponding scores.

### Receiver operating characteristic curve analysis

The ROC curve is generated by plotting the true positive rates of cutoff values on the y-axis against their false positive rates on the x-axis. The diagnostic accuracy of IL-41 in HCC was compared to that of existing pathology criteria. The curves of subjects were plotted, and the area under the curve (AUC) was determined.

### Statistical analysis

Measurement data are compared using the chi-square test or Fisher’s exact test. Factors showing statistical significance were analyzed by one-way ANOVA, logistic regression, and multivariate analyses to determine their correlations with IL-41 level, tumor recurrence and clinicopathological features of patients. Count data were compared using the Student’s *t*-test, and correlation between IL-41 and AFP was determined by the Spearman’s rank correlation coefficient. Survival of patients with different IL-41 expression levels was estimated by the Kaplan-Meier plot. All data analyses were performed in IBM SPSS v.28.0, and data were visualized using GraphPad Prism 7.0. A *P <*0.05 was considered statistically significant.

## Results

### IL-41 is highly expressed in the serum of AFP-negative HCC patients

**Figure 1:**
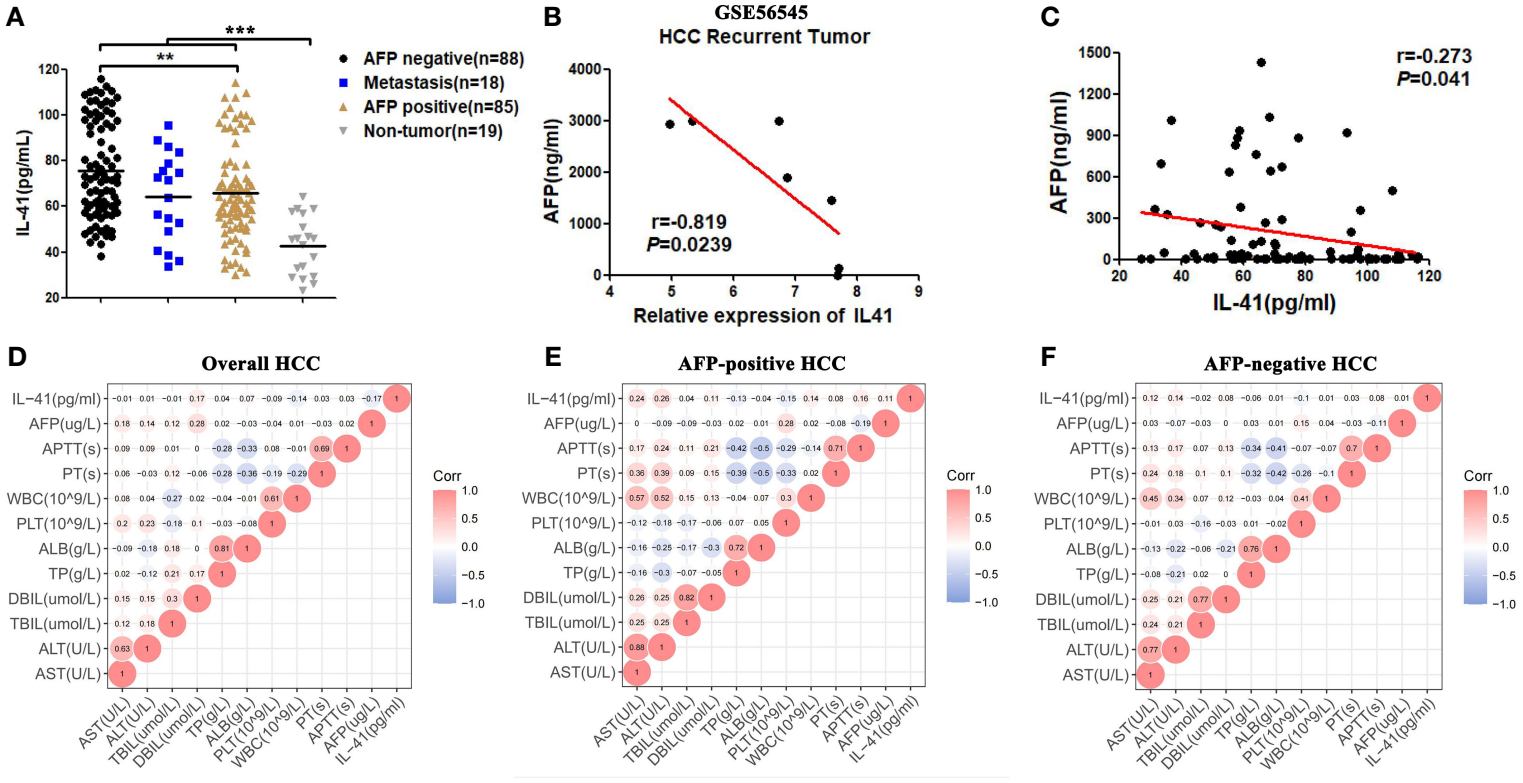
**(A)** The expression level of IL41 in serum of AFP negative, AFP positive HCC patients, liver metastatic cancer patients and healthy people(pg/ml) (**P<0.01, ***P<0.001); **(B)** Correlation between serum AFP(ng/ml) and tissue IL41 mRNA relative expression in patients with recurrence HCC in GSE56545 dataset (*P*<0.05); **(C)** Based on the data analysis of our center, serum IL41 of HCC patients with AFP no more than 1500ng/ml was significantly negatively correlated with AFP(*P*<0.05); **(D)** The serum indicators of all HCC patients included IL41, AST, ALT, TBIL, DBIL, TP, ALB, PT, APTT, AFP, WBC and PLT correlation heat map; **(E)** The serum indexes of AFP positive HCC patients included IL41, AST, ALT, TBIL, DBIL, TP, ALB, PT, APTT, AFP, WBC and PLT correlation heat map; **(F)** The serum markers of AFP negative HCC patients included correlation heat maps of IL41, AST, ALT, TBIL, DBIL, TP, ALB, PT, APTT, AFP, WBC and PLT.

We have previously screened the The Cancer Genome Atlas(TCGA) and Gene Expression Omnibus(GEO) databases and identified IL-41 as a potential gene associated with serum AFP expression in HCC patients. Although the TCGA dataset showed no significant difference between the relative tissue expression of IL-41 and AFP in HCC patients (**Supplementary Figure S1A**), we gradually observed a trend of increased negative correlation after narrowing the distribution range of AFP (data not shown). In order to assess the performance of IL-41 as a potential serum biomarker, we first measured the level of serum IL-41 expression in HCC patients before surgery (HCC was pathologically confirmed after resection), CRC patients with liver metastases (confirmed after colon or rectal cancer surgery), and healthy controls who underwent physical examination at our hospital around the same time period. ELISA showed that serum IL-41 expression was significantly higher in AFP-negative HCC patients than in AFP-positive HCC patients, CRC patients with liver metastases, and healthy controls (*P* < 0.01; **Figure 1A**). In addition, we found a GEO dataset showing a significant negative correlation between tissue IL-41 expression and serum AFP expression in relapsed HCC patients (**Figure 1B**) but not in relapse-free HCC patients (**Supplementary Figure S1B**). Next, we analyzed the correlation between the serum expression levels of AFP and IL-41 using data from a single-center study and found no significant correlation between the two markers, which was likely attributed to the large variance in AFP values. As a result, we continued to narrow the range of AFP values and discovered that serum IL-41 level was negatively correlated with AFP level when the latter was less than or equal to 1500 ng/mL (**Figure 1C**). Taken together, these results demonstrated that IL-41 may be a novel marker for HCC patients with low AFP expression.

Considering the prevalence of liver cancer secondary to hepatitis and cirrhosis in China, we sought to determine the correlation between IL-41 level and serum indicators of liver function and systemic inflammatory response. Our data revealed that IL-41 was not correlated with liver function indicators in all HCC patients (**Figure 1D**). To test the diagnostic sensitivity and specificity of IL-41, we also examined the serum expression level of IL-41 in patients with acute hepatitis (including sera of patients with HBV, HCV, and autoimmune hepatitis) who were positive for AFP. We found that serum IL-41 level varied greatly among patients with acute hepatitis and was comparable among the different groups of patients (**Supplementary Figure S1C**). Analysis of sera from AFP-negative and AFP-positive HCC patients demonstrated that serum IL-41 level was positively correlated with ALT and AST levels in AFP-positive HCC patients (**Figures 1D–F**, **Supplementary Figures S1D, E**) but not in AFP-negative HCC patients. Collectively, these data indicate that serum IL-41 level is significantly higher in HCC patients than in healthy individuals, and higher in AFP-negative than in AFP-positive HCC patients. In addition, serum IL-41 expression is not correlated with common liver function indicators in AFP-negative HCC patients.

### IL-41 is significantly upregulated in HCC tissues

**Figure 2:**
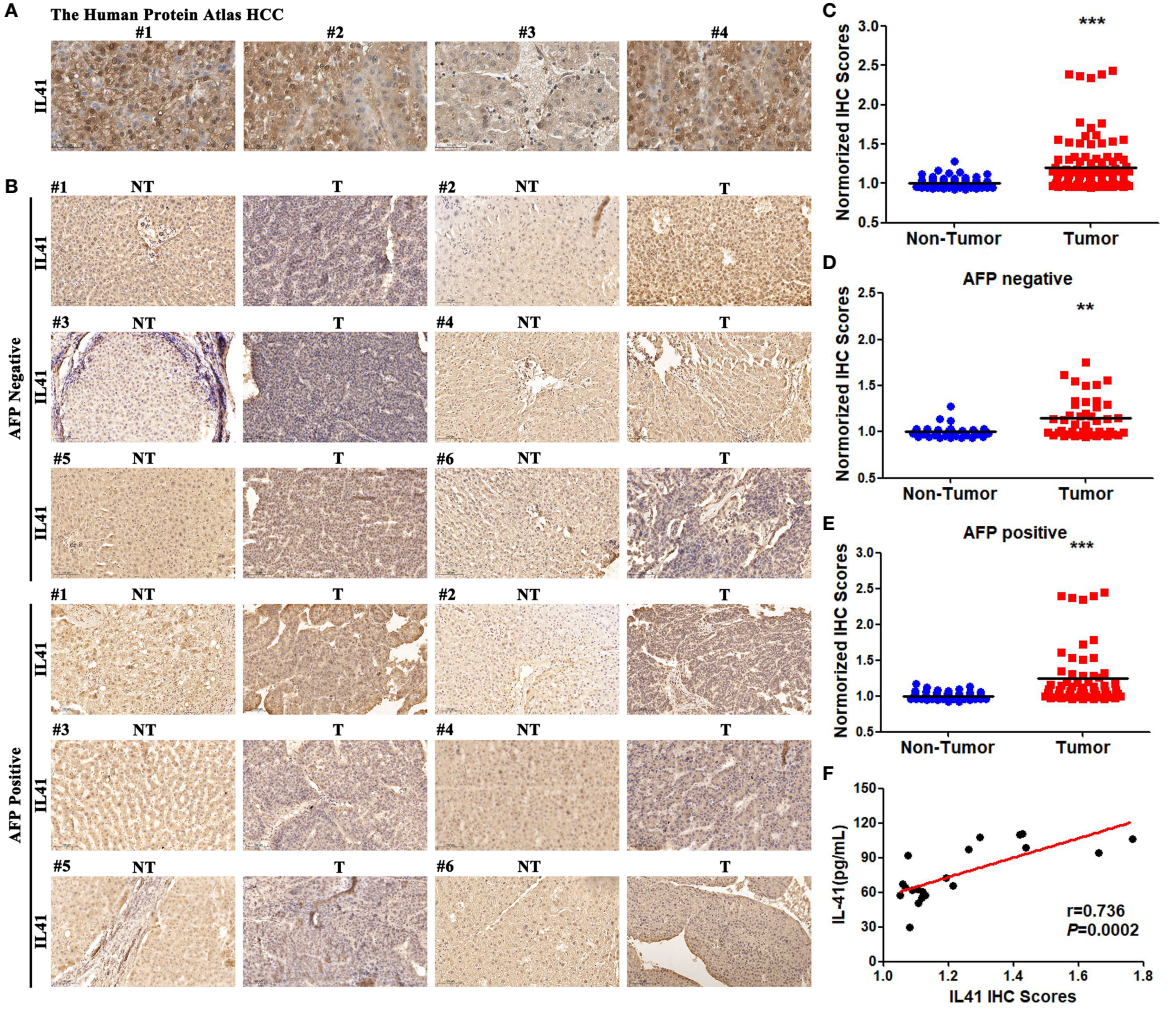
**(A)** IHC results of IL41 in Human Protein Atlas in HCC tissues; **(B)** IHC results of IL41 of 22 pairs of HCC analyzed by our center and 6 pairs of AFP positive and AFP negative HCC patients selected from corresponding paracancer tissues; **(C)** IL41 was significantly higher in HCC tissues than in paracancer tissues in all HCC patients (***P<0.001); **(D)** IL41 in HCC patients with negative AFP was significantly higher than that in paracancer tissues (**P<0.01). **(E)** IL41 in HCC patients with positive AFP was significantly higher than that in paracancer tissues (***P<0.001). **(F)** The expression of IL41 in 22 HCC tissues was significantly positively correlated with the serum IL14 level (*P*<0.01).

We next analyzed the relative expression of IL-41 in 22 pairs of HCC and adjacent tissues using the HPA database and by performing IHC staining of single-center tissue samples. Our results showed that HCC tissues were stained positive for IL-41 whereas intrahepatic cholangiocarcinoma (ICC) tissues were mostly negative for IL-41 (**Figure 2A**, ICC data not shown). IHC scoring of 12 pairs of tissue from AFP-positive (6 pairs) and AFP-negative (6 pairs) patients revealed higher IL-41 expression in HCC tissues (all patients or AFP negative or positive patients) than in paracancerous tissues (**Figures 2B–E**). Furthermore, we found that serum IL-41 expression was also positively correlated with tissue IL-41 expression in HCC (**Figure 2F**).

### High serum IL-41 expression is correlated with tumor recurrence and poor prognosis

**Figure 3:**
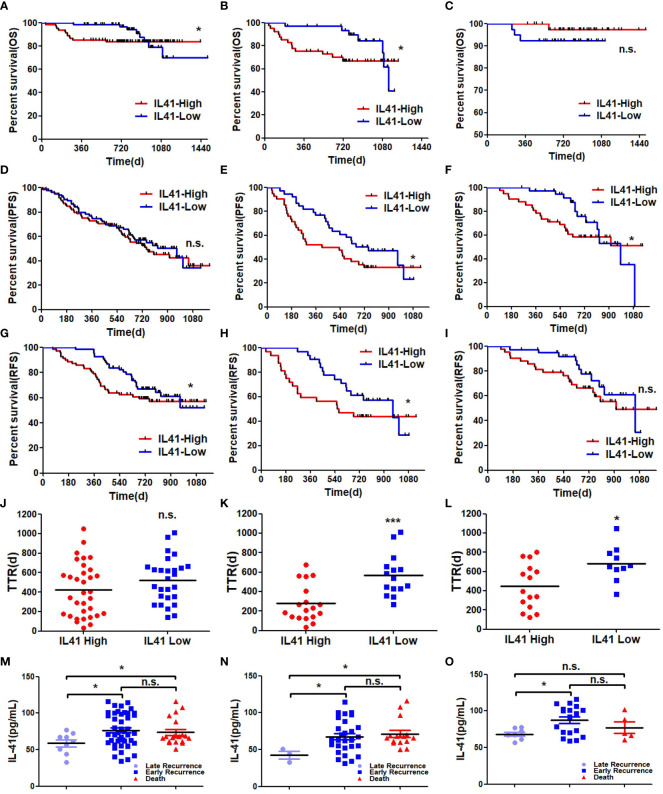
**(A)** OS of all HCC patients associated with high and low expression of IL41 (*P<0.05); **(B)** OS in AP-positive HCC patients associated with high and low expression of IL41 (*P<0.05); **(C)** OSin HCC patients with negative AFP associated with high and low expression of IL41 (n.s. no significance); **(D)** PFS (n.s, no significance) of all HCC patients associated with high and low expression of IL41; **(E)** PFS of AFP-positive HCC patients with high and low expression of IL41 (*P<0.05); **(F)** High and low expression of IL41 were associated with AFP negative PFS in HCC patients (*P<0.05); **(G)** RFS of all HCC patients associated with high and low expression of IL41 (*P<0.05); **(H)** RFS of AP-positive HCC patients associated with high and low expression of IL41 (*P<0.05); **(I)** RFS in HCC patients with negative AFP associated with high and low expression of IL41 (n.s. no significance); **(J)** TTR of all HCC patients associated with high and low expression of IL41 (n.s. no significance); **(K)** TTR of AP-positive HCC patients associated with high and low expression of IL41 (***P<0.001); **(L)** TTR of HCC patients with negative AFP associated with high and low expression of IL41 (*P<0.05); **(M)** The expression level of serum IL41 in HCC patients with late recurrence, early recurrence and death (*P<0.05, n.s. no significance); **(N)** The expression level of IL41 in serum of AFP positive HCC patients with late recurrence, early recurrence and death (*P<0.05, n.s. no significance); **(O)** Serum IL41 expression levels in AFP-negative HCC patients with late recurrence, early recurrence, and death (*P<0.05, n.s, no significance).

To further evaluate the diagnostic performance of IL-41, we analyzed the correlation between IL-41 expression and the various clinicopathological features of HCC patients. Our findings showed that serum IL-41 expression was significantly correlated with micro-vascular invasion(MVI), poorly differentiated cancer cells, and high preoperative AFP (**Table 1**). We further analyzed correlations between clinicopathologic characteristics and survival or recurrence of hepatocellular carcinoma patients. Tables listed factors such as serum AFP and IL-41 expression, and MVI had strong correlation between survival and recurrence (**Supplementary Tables S1**-**S3**). However, only AFP could be the sole risk for HCC patients survival (**Supplementary Table S4**). Besides, IL-41 could be one of the HCC recurrence and early recurrence predictors after resections (**Tables 2**, **3**). Moreover, we examined the OS, PFS, RFS, and postoperative recurrence of HCC patients with high and low IL-41 expression as well as HCC patients who were positive or negative for AFP. We found that OS was shorter in all HCC patients with high IL-41 expression and AFP-positive HCC patients (**Figures 3A, B**) but was similar between AFP-negative HCC patients with high or low IL-41 expression (**Figure 3C**). In addition, PFS was not significantly different among all HCC patients (**Figure 3D**) but was shorter in AFP-negative or -positive HCC patients with high serum IL-41 levels (**Figures 3E, F**). Since the patients included in this study had undergone resection for HCC, we also analyzed important indicators of post-resection outcomes, including RFS and TTR. RFS analysis showed that post-resection recurrence rate was higher in all HCC patients and AFP-positive HCC patients with high serum IL-41 expression (**Figures 3G, H**), but comparable between AFP-negative HCC patients with high or low IL-41 expression (**Figure 3I**). Interestingly, while there was no significant difference in TTR between the high and low IL-41 expression groups (**Figure 3J**), TTR was shorter in both the AFP-positive and AFP-negative subgroups with high IL-41 expression (**Figures 3K, L**). In summary, high expression of IL-41 is associated with poor differentiation, AFP, and suggests poor prognosis and post-resection recurrence. More importantly, we found that the serum IL-41 level was lower in patients with late recurrence (2 years after resection) than in patients with early recurrence and death (**Figures 3M–O**). These findings suggest that IL-41 expression may be a reliable predictor for tumor progression and survival outcomes in HCC patients.

**Table 1:** Correlation between the clinicopathologic characteristics and IL41 serum expression in hepatocellular carcinoma.

Clincopathological Features	Cases (n=162)	IL41 Serum Expression		*P* value
		IL41 ^high^ (n=81)	IL41^low^ (n=81)	
Narrow Surgical Edge (≤0.5cm)				
Yes	65	37	28	0.149
No	97	44	53	
Capsule Invasion				
Yes	52	27	25	0.736
No	110	54	56	
HBV Infection				
Yes	146	74	72	0.598
No	16	7	9	
Serum AFP before Resection (ng/ml)				
AFP positive	78	31	47	**0.012**
AFP negative	84	50	34	
Tumor Diameter(cm)				
≥ 5	52	31	21	0.092
< 5	110	50	60	
Tumor number				
≥ 2	18	12	6	0.134
< 2	144	69	75	
Age				
≥ 65	53	29	24	0.503
< 65	109	52	57	
Gender				
Male	113	60	53	0.231
Female	49	21	28	
MVI				
M0	47	4	43	**<0.001**
M1 or M2	115	77	38	
Edmondson-Steiner grading				
I+II	112	46	66	**0.001**
III+IV	50	35	15	

Bold font statistically significant.

**Table 2:** Univariate and multivariate cox hazard analysis of clinical features for HCC recurrence.

Clincopathological Features	Univariate analysis		*P* value	Multivariate analysis		*P* value
	HR	95 CI		HR	95 CI	
IL41(high)	2.787	1.440-5.398	**0.002**	1.858	1.389-3.889	**0.043**
MVI(M1/M2)	7.973	2.943-21.602	**<0.001**	7.173	2.305-22.322	**0.001**
Edmondson-Steiner grading(poor)	1.871	0.948-3.695	0.071	1.231	0.577-2.626	0.592
Narrow Surgical Edge	2.274	1.184-4.365	**0.014**	2.490	1.209-5.127	**0.013**

Bold font statistically significant.

CI, confidence interval; HR, Hazard ratio.

**Table 3:** Univariate and multivariate cox hazard analysis of clinical features for early HCC recurrence.

Clincopathological Features	Univariate analysis		*P* value	Multivariate analysis		*P* value
	HR	95 CI		HR	95 CI	
IL41(high)	0.19	0.053-0.681	**0.011**	5.250	1.467-18.784	**0.011**

Bold font statistically significant.

Abbreviations: CI, confidence interval; HR, Hazard ratio.

### The expression of IL-41 indicated malignant progression of HCC

**Figure 4:**
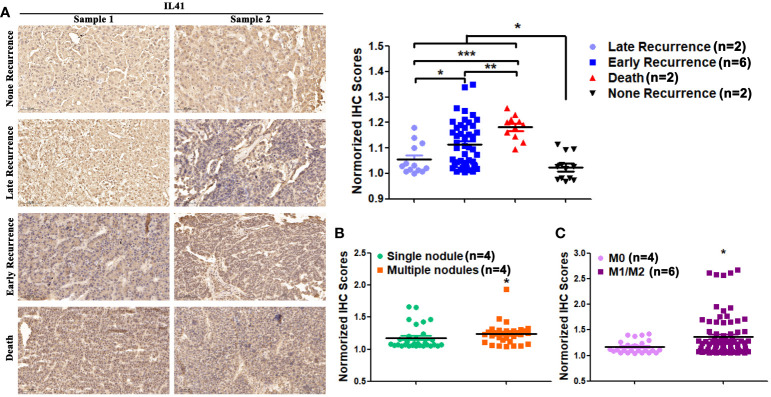
**(A)** The expression level of IL41 in tumor tissues of HCC patients with no recurrence, late recurrence, early recurrence and death (*P<0.05); **(B)** The expression level of IL41 in tumor tissues of patients with single and multiple HCC (*P<0.05, **P<0.01, ***P<0.001); **(C)** The expression level of IL41 in tumor tissues of HCC patients without microvascular invasion (M0) and with microvascular invasion (M1/2) (*P<0.05).

Since serum IL-41 expression is positively correlated with faster tumor progression and poor prognosis as well as tissue IL-41 expression, we also examined the relationship between tissue IL-41 expression and clinicopathological features. We measured IL-41 expression in HCC tissues of patients with no recurrence, early recurrence, late recurrence, and death after tumor resection. We found that IHC score was the lowest for patients without recurrence and highest for those who died, while the IHC score of patients with early recurrence was between those with late recurrence and death (**Figure 4A**). This suggests that there is a trend of worsened outcomes as the level of tissue IL-41 expression increased. Further analyses of tissue IL-41 expression and tumor number, tumor size, and microvascular invasion in the 22 HCC patients revealed that patients with multiple metastases and microvascular invasion had significantly higher tissue IL-41 expression level (**Figures 4B, C**). On the other hand, tumor size (tumor size ≥5 cm or <5cm) was not associated with tissue IL-41 expression (data not shown). In general, these findings indicate that high tissue IL-41 expression is associated with malignant pathological features and adverse tumor outcomes following surgical resection. We posit that postoperative detection of IL-41 expression not only aids in HCC diagnosis but also serves as a predictor for postoperative recurrence and mortality. Additionally, it can potentially act as a warning signal for more frequent follow-up and early preventive treatment in these patients.

### IL-41 can be used as a new serum diagnostic marker for HCC

**Figure 5:**
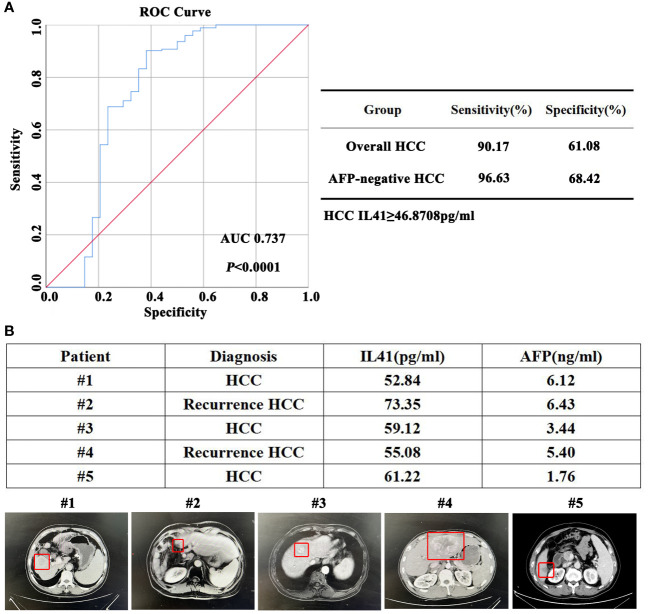
**(A)** ROC curve, sensitivity and specificity of IL41 in the diagnosis of HCC; **(B)** According to the diagnostic criteria in **(A)**, the diagnostic accuracy of serum IL41 in the diagnosis of HCC in 5 newly added AFP-negative HCC patients was 100% (HCC lesions marked in the red box below).

As described above, we analyzed the serum expression of IL-41 in 170 HCC patients, 18 patients with liver metastases (colon cancer liver metastasis), 15 patients with acute hepatitis, and 19 healthy controls. ROC curve analysis indicated that the IL-41 cutoff for HCC diagnosis was 46.87 pg/mL, and its diagnostic sensitivity and specificity were 90.17% and 61.08%, respectively (**Figure 5A**). It should be noted that the diagnostic sensitivity and specificity of IL-41 were 96.63% and 68.42% in AFP-negative HCC patients, respectively. The sensitivity of IL-41 for HCC diagnosis exceeds that of any single serological marker currently available. In order to further verify the reliability of IL-41, we expanded our cohort to include obese patients and liver cancer patients diagnosed with HCC by imaging but not confirmed by pathology. However, due to the limited number of AFP-negative patients in this expanded cohort, we found that serum IL-41 expression was higher than the diagnostic cutoff (46.87) in five newly added patients but lower in 2 patients with hepatic hemangioma (not shown), resulting in a diagnostic accuracy of 100% among these newly added liver cancer patients (**Figure 5B**). In summary, IL-41 is a promising sensitive serum marker for HCC diagnosis worthy of further research and application.

## Discussion

In the present study, we identified IL-41 as a potential diagnostic biomarker through database screening and verified its expression in clinical samples. Our data showed that high IL-41 expression was associated with early postoperative recurrence and poor prognosis in AFP-negative HCC patients. In addition, serum IL-41 expression was significantly higher in AFP-negative HCC patients than in AFP-positive HCC patients, colorectal cancer (CRC) patients with liver metastases, and healthy control. Furthermore, IL-41 expression in HCC tissues was higher in patients with early post-resection recurrence and death and was potentially associated with multiple metastases and microvascular invasion. Subsequent ROC curves and supplementation of additional patient sera confirmed the high sensitivity of IL-41 in the diagnosis of HCC, especially in AFP negative HCC patients. Taken together, our findings demonstrate that serum and tissue IL-41 expression is a promising new diagnostic marker for AFP-negative HCC.

HCC is a life-threatening and recurrent disease that impacts over a million patients worldwide every year (1), and its prevalence has been steadily rising in China due to the increasing rate of hepatitis B virus infection, making China a high-risk region for hepatitis and liver cancer (2). The discovery of new and reliable diagnostic and therapeutic targets for HCC is key to defeating liver cancer. Although AFP (3), PIVKA2 (4), GP73 (5), and other proteins have been identified as biomarkers for liver cancer diagnosis and treatment, there is currently no reliable tumor marker for patients with AFP-negative HCC. In this study, we retrospectively analyzed the clinical data of 176 HCC patients and identified IL-41 as a potential marker that negatively correlates with AFP expression using the TCGA the GEO databases and the biobank data of our center. The excellent diagnostic sensitivity of IL-41 (90.17%) indicates that it is the best serum marker thus far for HCC diagnosis, and the first and most sensitive (96.63%) marker for AFP-negative HCC. However, it is undeniable that IL-41 has limited specificity for HCC diagnosis, which may be attributed to the complex functions of the cytokine.

IL-41, also known as METRNL, is a novel secreted immunomodulatory protein highly expressed in barrier tissues, especially white adipose tissue (6, 7). This cytokine was first reported in 2004 (8) and its expression has been shown to be upregulated by factors such as inflammation (9), exercise, and cold exposure. The function of IL-41 is complex and diverse. It can act as a neurotrophic factor for neurons, neuroblasts, and spiral ganglion neurons (10), attenuate lipid-induced inflammation, alleviate insulin resistance, and lead to increased energy consumption and improved glucose tolerance in mice by inducing the browning of white fat (11). The role of IL-41 in inflammatory response is a key focus of current research. IL-41 has been reported to be significantly upregulated in inflammatory bowel disease, sepsis, psoriatic arthritis, and other inflammatory diseases, and plays a protective role in local tissues (12–14). Therefore, IL-41 is a potential diagnostic marker and therapeutic target for a wide range of immune diseases and may impact the progression of liver cancer. The 15 newly added hepatitis patients were active hepatitis patients with elevated AFP expression, and we found that serum IL-41 expression significantly varied among hepatitis patients. On the other hand, serum IL-41 expression was positively correlated with ALT and AST levels in AFP-positive liver cancer patients. Therefore, liver inflammation and acute liver damage caused by active hepatitis can potentially lead to inaccurate diagnosis by IL-41. Similar to the need of excluding active hepatitis, pregnancy, and reproductive system tumors when diagnosing liver cancer with AFP, active hepatitis and acute liver damage should also be excluded for IL-41-based liver cancer diagnosis. In addition, the serum levels of IL-41 in patients with autoimmune diseases, allergies, and infectious diseases such as chronic obstructive pulmonary disease in elderly patients with liver cancer, other types of viral hepatitis, and very rare liver cancer with liver abscess, should also be investigated to determine the effects of these conditions on IL-41 expression. We are currently measuring the serum level of IL-41 in obese patients and additional multicenter liver cancer patients to obtain a more scientific and accurate diagnostic cutoff for IL-41 in liver cancer.

We must note that the correlation analysis results of IL-41 and AFP are partly contradictory and the mRNA level of IL-41 in the TCGA database is significantly lower than that in normal liver tissues. During our search for diagnostic markers, we originally planned to look for extracellular proteins that are significantly negatively associated with AFP level but were constrained by the limited datasets that also examined serum AFP expression levels. Nevertheless, we still identified the GSE56545 dataset and found that the serum AFP expression was significantly negatively correlated with tissue IL-41 level in HCC patients who relapsed after resection. However, our analyses of the TCGA dataset and single-center data showed no correlation between serum AFP level and serum or tissue IL-41 expression. Considering the large variance in AFP expression, data with greater than 10,000 or even 100,000 ng/mL seriously affected the significance of the correlation. It is worth mentioning that the negative correlation between serum AFP and serum IL-41 increased as AFP decreased. In fact, when AFP was ≤ 1500ng/mL, AFP was negatively correlated with serum IL-41 expression. Furthermore, the IL-41 mRNA level in the TCGA dataset was lower or similar in HCC tissues compared with normal tissues, which resulted in no difference in serum or tissue IL-41 protein expression. Since proteins are responsible for mediating functions and there are various mechanisms of transcriptional modifications that can affect protein function and stability, the presence or absence of differences in mRNA expression is unlikely to affect the role of IL-41 protein in the diagnosis and treatment of HCC.

Similarly, the important diagnostic role of IL-41 is evidenced in the poor prognosis of HCC patients. Analyses of OS, PFS, RFS, and TTR of HCC patients with high and low IL-41 expression revealed that high serum IL-41 expression was associated with poor prognosis and post-resection recurrence in HCC patients. IHC analysis also indicated that high tissue IL-41 expression is associated with early recurrence, death, multiple metastases, and microvascular invasion after HCC resection. However, the source of IL-41 and the molecular mechanisms by which IL-41 overexpression promotes malignant progression of liver cancer are still unknown. Therefore, further studies are warranted to answer these questions. Our supplementary data hinted that IL-41 is associated with various immune cells, most strongly with macrophages. We used CD68 to locate macrophages in HCC tissues and stain the markers of M2 macrophages in order to explore whether macrophages and other cells in the tumor microenvironment are involved in the high expression of IL-41 and the malignant progression of HCC. Our team plans to send to HCC tissues with significantly high and low expression of IL-41, as well as transcriptome sequencing of HCC cell lines with overexpression and knockdown of IL-41, to search for potential downstream pathways and targets of IL-41.

In summary, IL-41 is a promising novel serum marker for HCC diagnosis and a potential predictor for poor prognosis and malignant progression of HCC. This marker is the cornerstone that paves the way for the development of further diagnostic markers and therapeutic targets for AFP-negative liver cancer patients.

## Data availability statement

The datasets presented in this study can be found in online repositories. The names of the repository/repositories and accession number(s) can be found in the article/**Supplementary Material**.

## Ethics statement

The studies involving humans were approved by the Ethics Committee of Xi’an Jiaotong University. The studies were conducted in accordance with the local legislation and institutional requirements. Written informed consent for participation in this study was provided by the participants’ legal guardians/next of kin. Written informed consent was obtained from the individual(s) for the publication of any potentially identifiable images or data included in this article.

## Author contributions

BY: Data curation, Funding acquisition, Investigation, Resources, Supervision, Writing – review & editing. YL: Funding acquisition, Data curation, Investigation, Resources, Writing – original draft, Writing – review & editing. HW: Investigation, Methodology, Software, Writing – original draft. DR: Investigation, Resources, Data curation, Writing – original draft. JL: Investigation, Methodology, Resources, Writing – original draft. ZM: Investigation, Methodology, Writing – original draft. CL: Investigation, Methodology, Writing – original draft. YoH: Investigation, Software, Writing – original draft. JZ: Investigation, Software, Writing – original draft. RF: Methodology, Writing – original draft. JY: Methodology, Software, Writing – original draft. JS: Methodology, Writing – original draft. YiH: Resources, Writing – original draft.

## Supplementary material

Supplementary Figure 1
**(A)** There was no significant correlation between serum AFP of HCC patients and tissue IL41 mRNA in TCGA database; **(B)** There was no correlation between the relative expression of IL41 mRNA in non-recurrent HCC tissues and serum AFP in GSE56545 dataset; **(C)** There were 15 new patients with acute hepatitis and the expression of IL41 in different groups was analyzed. There was no significant difference in serum IL41 between hepatitis patients and other groups; (***P*<0.01,****P*<0.001), n.s.: no signifcance. **(D)** There was a significant positive correlation between serum IL41 and ALT in AFP positive HCC patients(*P*<0.05). **(E)** There was a significant positive correlation between serum IL41 and AST in AFP positive HCC patients(*P*<0.05). **(F)** IL41(METRNL) in the TCGA database was positively correlated with multiple immune cell infiltrations (****P*<0.001).

Supplementary Figure 2
**(A)** The OS of HCC patients with high or low expression of IL41 presented in the TCGA database (*P*<0.05); **(B)** The OS of HCC patients with high or low IL41 expression combined with hepatitis B was shown in TCGA database(*P*<0.05); **(C)** The OS of HCC patients with high or low IL41 expression combined with non-alcoholic hepatitis was shown in the TCGA database (*P*<0.05); **(D)** OS of HCC patients with positive or negative AFP in our center (****P*<0.001).
