# Supplementary figures and images for: Interleukin-41: a novel serum marker for the diagnosis of alpha-fetoprotein-negative hepatocellular carcinoma
Source: Front Oncol. 2024 May 21;14:1408584. doi: 10.3389/fonc.2024.1408584 (PMC11148433; doi:10.3389/fonc.2024.1408584)

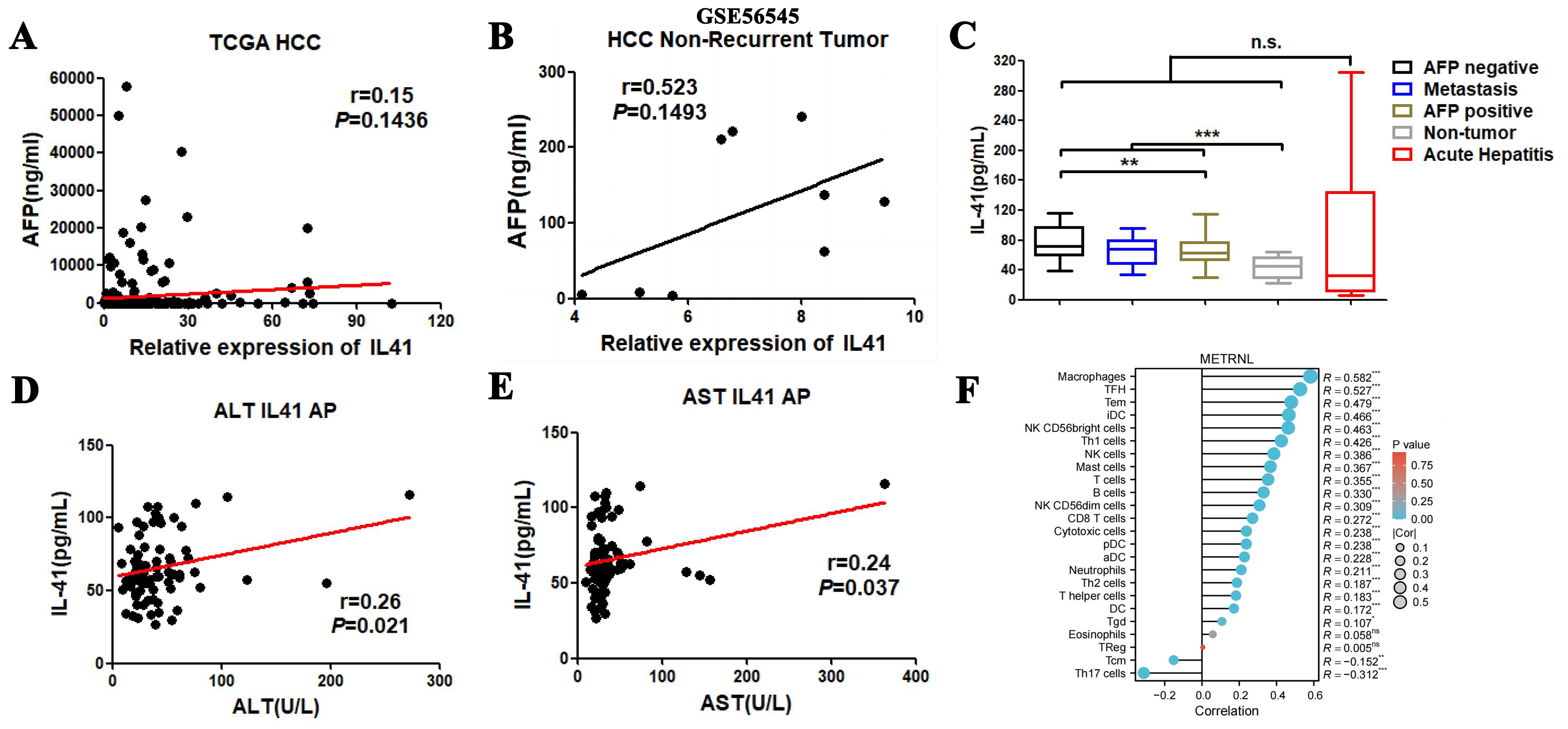

Supplement: Supplementary Figure 1 — (A) There was no significant correlation between serum AFP of HCC patients and tissue IL41 mRNA in TCGA database; (B) There was no correlation between the relative expression of IL41 mRNA in non-recurrent HCC tissues and serum AFP in GSE56545 dataset; (C) There were 15 new patients with acute hepatitis and the expression of IL41 in different groups was analyzed. There was no significant difference in serum IL41 between hepatitis patients and other groups; (**P<0.01,***P<0.001), n.s.: no signifcance. (D) There was a significant positive correlation between serum IL41 and ALT in AFP positive HCC patients(P<0.05). (E) There was a significant positive correlation between serum IL41 and AST in AFP positive HCC patients(P<0.05). (F) IL41(METRNL) in the TCGA database was positively correlated with multiple immune cell infiltrations (***P<0.001). [file Image_1.tif]

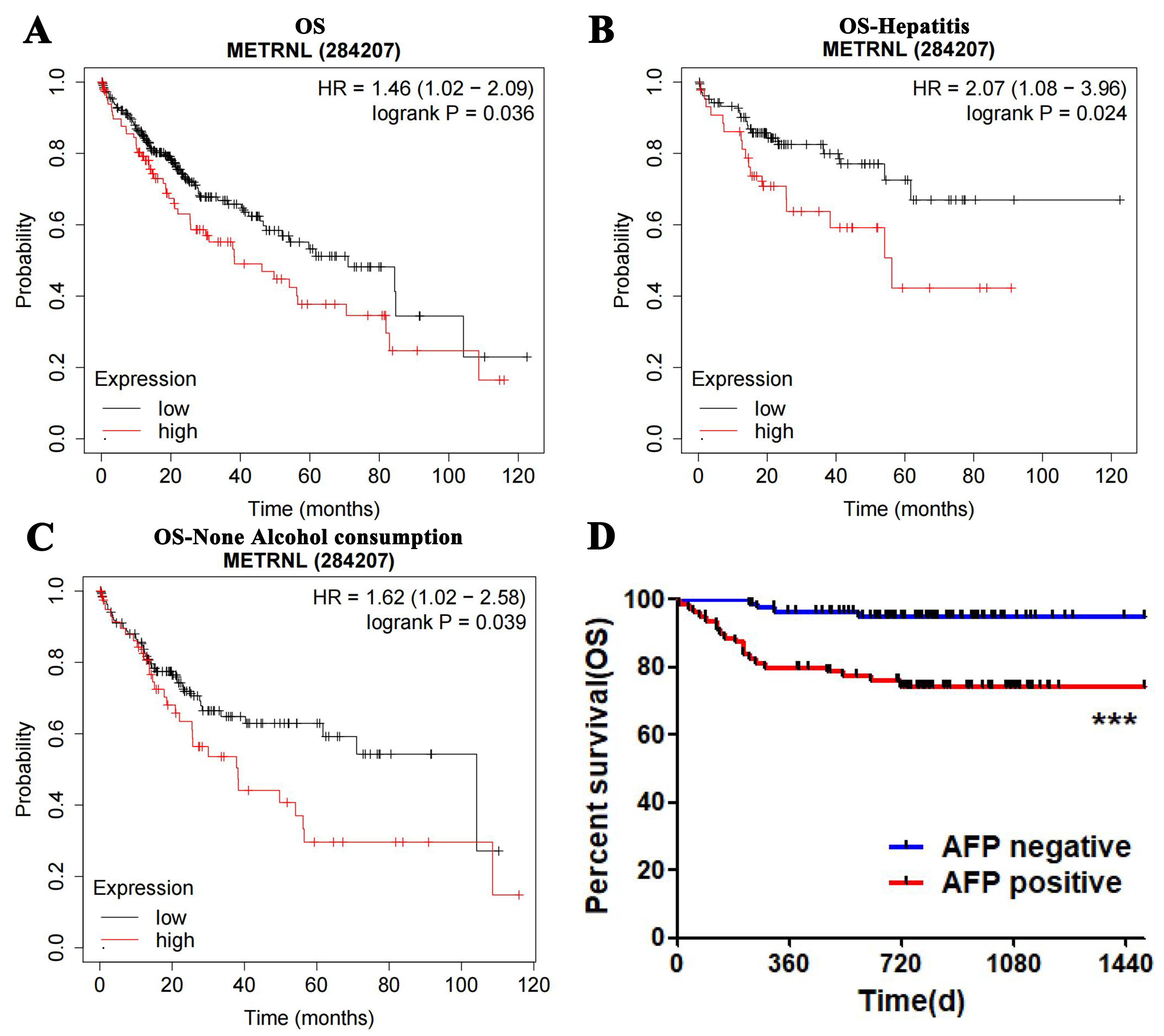

Supplement: Supplementary Figure 2 — (A) The OS of HCC patients with high or low expression of IL41 presented in the TCGA database (P<0.05); (B) The OS of HCC patients with high or low IL41 expression combined with hepatitis B was shown in TCGA database(P<0.05); (C) The OS of HCC patients with high or low IL41 expression combined with non-alcoholic hepatitis was shown in the TCGA database (P<0.05); (D) OS of HCC patients with positive or negative AFP in our center (***P<0.001). [file Image_2.tif]
