# Supplementary material for: Interleukin-41: a novel serum marker for the diagnosis of alpha-fetoprotein-negative hepatocellular carcinoma
Source: Front Oncol. 2024 May 21;14:1408584. doi: 10.3389/fonc.2024.1408584 (PMC11148433; doi:10.3389/fonc.2024.1408584)
Supplement: Supplementary file 3 [file Table_1.docx]

Table S1.Correlation between the clinicopathologic characteristics and survival of hepatocellular carcinoma patients

| Clincopathological Features | Cases  (n=162) | Survival | | *P* value |
| --- | --- | --- | --- | --- |
|  |  | Death(n=20) | Live(n=142) |  |
| Narrow Surgical Edge  (≤0.5cm) |  |  |  |  |
| Yes | 65 | 5 | 60 | 0.141 |
| No | 97 | 15 | 82 |  |
| Capsule Invasion |  |  |  |  |
| Yes | 52 | 4 | 48 | 0.216 |
| No | 110 | 16 | 94 |  |
| IL41 serum expression  (pg/ml) |  |  |  |  |
| IL41 ^high^ | 81 | 12 | 69 | 0.339 |
| IL41 ^low^ | 81 | 8 | 73 |  |
| HBV Infection |  |  |  |  |
| Yes | 146 | 18 | 128 | 1.000 |
| No | 16 | 2 | 14 |  |
| Serum AFP before Resection  (ng/ml) |  |  |  |  |
| AFP positive | 78 | 15 | 63 | **0.010** |
| AFP negative | 84 | 5 | 79 |  |
| Tumor Diameter(cm) |  |  |  |  |
| ≥ 5 | 52 | 10 | 42 | 0.067 |
| < 5 | 110 | 10 | 100 |  |
| Tumor number |  |  |  |  |
| ≥ 2 | 18 | 3 | 15 | 0.833 |
| < 2 | 144 | 17 | 127 |  |
| Age |  |  |  |  |
| ≥ 65 | 51 | 6 | 45 | 0.879 |
| < 65 | 111 | 14 | 97 |  |
| Gender |  |  |  |  |
| Male | 113 | 15 | 98 | 0.585 |
| Female | 49 | 5 | 44 |  |
| MVI |  |  |  |  |
| M0 | 47 | 2 | 45 | **0.045** |
| M1 or M2 | 115 | 18 | 97 |  |
| Edmondson-Steiner grading |  |  |  |  |
| Ⅰ+Ⅱ | 112 | 9 | 103 | **0.013** |
| Ⅲ+Ⅳ | 50 | 11 | 39 |  |

Note: Bold font statistically significant.
