# Supplementary material for: Interleukin-41: a novel serum marker for the diagnosis of alpha-fetoprotein-negative hepatocellular carcinoma
Source: Front Oncol. 2024 May 21;14:1408584. doi: 10.3389/fonc.2024.1408584 (PMC11148433; doi:10.3389/fonc.2024.1408584)
Supplement: Supplementary file 4 [file Table_2.docx]

Table S2.Correlation between the clinicopathologic characteristics and recurrence of hepatocellular carcinoma patients

| Clincopathological Features | Cases  (n=162) | Recurrence | | *P* value |
| --- | --- | --- | --- | --- |
|  |  | Yes(n=61) | No(n=101) |  |
| Narrow Surgical Edge  (≤0.5cm) |  |  |  |  |
| Yes | 65 | 32 | 33 | **0.013** |
| No | 97 | 29 | 68 |  |
| Capsule Invasion |  |  |  |  |
| Yes | 52 | 17 | 35 | 0.370 |
| No | 110 | 44 | 66 |  |
| IL41 serum expression  (pg/ml) |  |  |  |  |
| IL41 ^high^ | 81 | 40 | 41 | **0.002** |
| IL41 ^low^ | 81 | 21 | 60 |  |
| HBV Infection |  |  |  |  |
| Yes | 146 | 57 | 89 | 0.271 |
| No | 16 | 4 | 12 |  |
| Serum AFP before Resection  (ng/ml) |  |  |  |  |
| AFP positive | 78 | 32 | 46 | 0.393 |
| AFP negative | 84 | 29 | 55 |  |
| Tumor Diameter(cm) |  |  |  |  |
| ≥ 5 | 52 | 19 | 33 | 0.840 |
| < 5 | 110 | 42 | 68 |  |
| Tumor number |  |  |  |  |
| ≥ 2 | 18 | 9 | 9 | 0.252 |
| < 2 | 144 | 52 | 92 |  |
| Age |  |  |  |  |
| ≥ 65 | 51 | 16 | 35 | 0.262 |
| < 65 | 111 | 45 | 66 |  |
| Gender |  |  |  |  |
| Male | 113 | 45 | 68 | 0.387 |
| Female | 49 | 16 | 33 |  |
| MVI |  |  |  |  |
| M0 | 47 | 5 | 42 | **<0.001** |
| M1 or M2 | 115 | 57 | 59 |  |
| Edmondson-Steiner grading |  |  |  |  |
| Ⅰ+Ⅱ | 112 | 37 | 75 | 0.069 |
| Ⅲ+Ⅳ | 50 | 24 | 26 |  |
