# Supplementary material for: Interleukin-41: a novel serum marker for the diagnosis of alpha-fetoprotein-negative hepatocellular carcinoma
Source: Front Oncol. 2024 May 21;14:1408584. doi: 10.3389/fonc.2024.1408584 (PMC11148433; doi:10.3389/fonc.2024.1408584)
Supplement: Supplementary file 5 [file Table_3.docx]

Table S3.Correlation between the clinicopathologic characteristics and recurrence time of hepatocellular carcinoma patients

| Clincopathological Features | Cases  (n=61) | Recurrence | | *P* value |
| --- | --- | --- | --- | --- |
|  |  | Early(n=47) | Late(n=14) |  |
| Narrow Surgical Edge  (≤0.5cm) |  |  |  |  |
| Yes | 32 | 23 | 9 | 0.313 |
| No | 29 | 24 | 5 |  |
| Capsule Invasion |  |  |  |  |
| Yes | 17 | 15 | 2 | 0.341 |
| No | 44 | 32 | 12 |  |
| IL41 serum expression  (pg/ml) |  |  |  |  |
| IL41 ^high^ | 40 | 35 | 5 | **0.018** |
| IL41 ^low^ | 21 | 12 | 9 |  |
| HBV Infection |  |  |  |  |
| Yes | 57 | 43 | 14 | 0.565 |
| No | 4 | 4 | 0 |  |
| Serum AFP before Resection  (ng/ml) |  |  |  |  |
| AFP positive | 32 | 25 | 7 | 0.834 |
| AFP negative | 29 | 22 | 7 |  |
| Tumor Diameter(cm) |  |  |  |  |
| ≥ 5 | 19 | 14 | 5 | 0.927 |
| < 5 | 42 | 33 | 9 |  |
| Tumor number |  |  |  |  |
| ≥ 2 | 9 | 8 | 1 | 0.627 |
| < 2 | 52 | 39 | 13 |  |
| Age |  |  |  |  |
| ≥ 65 | 16 | 13 | 3 | 0.905 |
| < 65 | 45 | 34 | 11 |  |
| Gender |  |  |  |  |
| Male | 45 | 34 | 11 | 0.905 |
| Female | 16 | 13 | 3 |  |
| MVI |  |  |  |  |
| M0 | 5 | 3 | 2 | 0.322 |
| M1 or M2 | 56 | 44 | 12 |  |
| Edmondson-Steiner grading |  |  |  |  |
| Ⅰ+Ⅱ | 37 | 28 | 9 | 0.751 |
| Ⅲ+Ⅳ | 24 | 19 | 5 |  |
