# Supplementary material for: Interleukin-41: a novel serum marker for the diagnosis of alpha-fetoprotein-negative hepatocellular carcinoma
Source: Front Oncol. 2024 May 21;14:1408584. doi: 10.3389/fonc.2024.1408584 (PMC11148433; doi:10.3389/fonc.2024.1408584)
Supplement: Supplementary file 6 [file Table_4.docx]

Table S4.Univariate and multivariate cox hazard analysis of clinical features for survival

| Clincopathological Features | Univariate analysis | | *P* value | Multivariate analysis | | *P* value |
| --- | --- | --- | --- | --- | --- | --- |
|  | HR | 95 CI |  | HR | 95 CI |  |
| AFP(AFP positive) | 3.762 | 1.297-10.911 | **0.015** | 3.494 | 1.164-10.490 | **0.026** |
| MVI(M1/M2) | 4.175 | 0.929-18.769 | 0.062 | 3.194 | 0.649-15.714 | 0.153 |
| Tumor size(≥5cm) | 2.381 | 0.923-6.142 | 0.073 | 1.943 | 0.719-5.247 | 0.19 |
| Edmondson-Steiner grading(poor) | 3.228 | 1.242-8.388 | **0.016** | 1.973 | 0.702-5.543 | 0.197 |
